# Oleic acid reduces steroidogenesis by changing the lipid type stored in lipid droplets of ovarian granulosa cells

**DOI:** 10.1186/s40104-021-00660-5

**Published:** 2022-02-08

**Authors:** Xiaoge Zhou, Zhaoyi Mo, Yankun Li, Liang Huang, Sihai Yu, Lan Ge, Yamei Hu, Shengjie Shi, Lutong Zhang, Liguang Wang, Lei Gao, Gongshe Yang, Guiyan Chu

**Affiliations:** 1 Key Laboratory of Animal Genetics, Breeding and Reproduction of Shaanxi Province, Yangling, 712100 China; 2 Laboratory of Animal Fat Deposition & Muscle Development, College of Animal Science and Technology, Northwest A&F University, Yangling, 712100 China; 3 College of Animal Science and Technology, Northwest A&F University, Yangling, 712100 China; 4 College of veterinary medicine, Northwest A&F University, Yangling, 712100 China

**Keywords:** Fertility, Granulosa cell, Lipid droplet, Oleic acid, Steroidogenesis

## Abstract

**Background:**

Oleic acid is an abundant free fatty acid present in livestock that are in a negative energy-balance state, and it may have detrimental effects on female reproduction and fertility. Oleic acid induces lipid accumulation in bovine granulosa cells, which leads to a foam cell-like morphology and reduced steroidogenesis. However, why oleic acid increases lipid accumulation but decreases steroidogenesis remains unclear. This study focused on oleic acid’s effects on lipid type and steroidogenesis.

**Results:**

Oleic acid increased the lipid accumulation in a concentration-dependent manner and mainly increased the triglyceride level and decreased the cholesterol ester level. Oleic acid also led to a decline in estradiol and progesterone production in porcine granulosa cells in vitro. In addition, oleic acid up-regulated the expression of *CD36* and diacylglycerol acyltransferase 2, but down-regulated the expression of 3-hydroxy-3-methylglutaryl-coenzyme A reductase, scavenger receptor class B member 1 and acetyl-Coenzyme A acetyltransferase 2, as well as steroidogenesis-related genes, including cytochrome P450 family 11 subfamily A member 1, cytochrome P450 family 19 subfamily A member 1 and 3 as well as steroidogenic acute regulatory protein at the mRNA and protein levels. An oleic acid-rich diet also enhanced the triglyceride levels and reduced the cholesterol levels in ovarian tissues of female mice, which resulted in lower estradiol levels than in control-fed mice. Compared with the control, decreases in estrus days and the numbers of antral follicles and corpora lutea, as well as an increase in the numbers of the atretic follicles, were found in the oleic acid-fed female mice.

**Conclusions:**

Oleic acid changed the lipid type stored in lipid droplets of ovarian granulosa cells, and led to a decrease in steroidogenesis. These results improve our understanding of fertility decline in livestock that are in a negative energy-balance state.

## Introduction

Lipid droplets (LDs) are specialized cellular organelles consisting of a neutral lipid core covered by a phospholipid monolayer embedded with various proteins [1]. The LDs mainly accumulate either triglycerides (TGs) or cholesteryl esters (CEs), depending on the tissue [2]. The cells in some metabolic tissues, such as adipose, liver and muscle, accumulate TGs [3], whereas steroidogenic cells in some tissues, such as granulosa cells in ovaries and Leydig cells in testes, mostly accumulate CEs [4, 5]. Compared with other tissues, LDs that have CE cores and are stored in steroidogenic tissues tend to be smaller and more numerous [6]. When loaded with different substrates, the lipid type stored in LDs within cells changes. Rat granulosa cells incubated with oleic acid and palmitic acid significantly accumulate TG in their LDs, but if loaded with HDL (high-density lipoprotein), then they accumulate CE in their LDs [7]. Thus, the lipid type stored in the LDs of granulosa cells changes when loaded with different substrates.

The levels of non-esterified fatty acids in follicular fluid increases when high-yielding dairy cows are in a negative energy-balance state owing to the mobilization of storage fat in adipose tissues [8, 9]. While beta-oxidation is necessary for oocyte development, excessive lipid levels may exert toxic effects on cellular functions (lipotoxicity), especially in cells of non-adipose tissues that have a limited lipid storage capacity [10, 11]. Oleic, palmitic and stearic acids are abundant free non-esterified fatty acids present in the blood and follicular fluids of pigs, cows and sheep [12, 13]. Many studies have focused on the harmful effects of palmitic and stearic acids, as a saturated fatty acid, on lipotoxicity [14, 15]. Additionally, the adverse effects of palmitic and stearic acids may be counteracted by oleic acid [9, 16]. The protective function of oleic acid is the induction of TG accumulation in cells [17, 18]. Although it plays a protective role in many physiological processes, oleic acid at a high level has adverse effects on female fertility. The 400 μmol/L oleic acid-induced accumulation of LDs in bovine granulosa cells, leads to a foam cell-like morphology and reduces the production of 17-beta-estradiol and progesterone. In addition, oleic acid increases the transcript levels of the fatty acid transporters *CD36* and solute carrier family 27 member 1, but obviously reduces the mRNA expression of cytochrome P450 family 11 subfamily A member 1 (*CYP11A1*) and other steroidogenic genes [19]. Exposure to 400 μmol/L oleate induces the down-regulation of the granulosa cell identity marker Forkhead box L2 (*FOXL2*), but induces the up-regulation of the Sertoli cell marker SRY-box transcription factor 9 in bovine granulosa cells [20].

Cholesterol is the raw material for steroid synthesis and is derived from a variety of sources, including lipoprotein uptake, endogenous cholesterol synthesis and release from LDs stored in cells [21]. Most importantly, Meikle et al. found that 700 μmol/L oleic acid significantly reduces testosterone levels by decreasing the cellular cholesterol content and inhibiting cholesteryl esterase activity in mouse Leydig cells [22]. Thus, oleic acid may negatively affect cholesterol metabolism in ovarian granulosa cells, but the exact mechanism used is not clear. Consequently, we hypothesized that oleic acid affects the lipid type stored in LDs of ovarian granulosa cells, which ultimately leads to a decrease in steroidogenesis. In the present study, we measured the levels of TGs and CEs, as well as production of estradiol and progesterone, after an oleic acid treatment. To further clarify the effects of a high level of oleate on reproductive performance, we added 20% sodium oleate to the diet of female mice and investigated the estrus cycle and follicular development. The results provide new insights into the mechanisms behind the fertility decline in livestock that are in a negative energy-balance state.

## Material and methods

### Animal and samples

Four-week-old C57BL/6 female mice were purchased from the Laboratory Animal Center of Xi’an Jiaotong University. The animals were housed in temperature- and light-controlled (21–22 °C, 12 h light-dark cycle) conditions with ad libitum access to water and customized diet (Xietong Pharmaceutical Bio-Engineering CO. LTD., Jiangsu, China). After a week of adaptation, mice (*n* = 60) were randomly divided into two groups: a cohort of mice (*n* = 30) was selected to give 20% sodium oleic acid (OA) (Cool chemistry, Beijing, China) and other cohorts (*n* = 30) received control diet as negative control (NC).

After 8 weeks of sodium oleic acid treatment, mice were sacrificed using ether. The blood, adipose tissue, livers and ovary were collected. All animal protocols were approved by the EAMC (Committee of Experimental Animal Management) at Northwest Agriculture and Forestry University, China.

### Porcine granulosa cells cultures and treatment

Fresh Landrace ovaries were isolated from local slaughterhouses, placed in physiological saline at 37 °C supplemented with penicillin and streptomycin, and brought back to the laboratory within 2 h. Follicular fluid was extracted from follicles with a diameter of 3 to 6 mm by needles, centrifuged at 1000 r/min for 10 min at room temperature, and then the supernatant was discarded. The pellets were resuspended in DMEM/F12 (cytiva, Shanghai, China) containing 10% fetal bovine serum (Gibco, ThermoFisher scientific, Shanghai, China), seeded in cell culture plates at 37 °C in humidified 5% CO_2_. After 24 h of culture, the cells were gently washed with phosphate-buffered saline (PBS) to remove other tissue and cell fragments. The granulosa cells were treated with 0.5% FA-free bovine serum albumin (Solarbio, Beijing, China) and oleic acid (Aladdin, Shanghai, China) for 4 d. The DMEM/F12 containing 0.5% FA-free bovine serum albumin as a control. The medium was changed every two days.

### Cell viability assay

The viability of granulosa cells was measured by a cell counting kit-8 (Dojindo, Japan) according to the manufacturer’s instructions. Cells were seeded in 96-well plate at a density of 2 × 10^3^ and cultured in 100 μL medium treated with different concentrations of oleic acid for 24 h. Then, 10 μL CCK-8 kit reagents and 90 μL medium were added to each well and incubated the cells at 37 °C for 2 h. Absorbance was measured at 450 nm using Multiskan TM FC (ThermoFisher scientific, Shanghai, China).

### Lipid profile assays

Mice were fasted overnight. Serum samples were collected from angular vein blood and kept in − 80 °C. The concentration of TG, CE, HDL and LDL in serum were detected by Chemray 800 Automatic biochemical analyzer (Servicebio, Wuhan, China). The TG concentration of the cells as well as the ovary was measured by a TG content detection kit (Nanjing Jiancheng Bioengineering Institute, Nanjing, China). And the total cholesterol content detection kit (Nanjing Jiancheng Bioengineering Institute, Nanjing, China) was used to detect the total cholesterol content of the cells and the ovary.

### Estradiol and progesterone measurement

Porcine granulosa cells were treated with different concentrations of oleic acid for 4 d, then culture medium was collected to store at − 80 °C. The concentration of estradiol and progesterone was detected with ELISA assay kit (Mlbio, Shanghai, China). There were triplicates for each measure. The absorbance at 450 nm represents the relative concentration of hormones using Multiskan TM FC (Thermo scientific, Shanghai, China). In addition, the serum levels of estradiol (F2566-B), progesterone (F2568-B) and FSH (Follicle-stimulating hormone) (F2555-B) of female mice were measured using the ELISA kit (Shanghai FANKEL Industrial Co., Ltd., Shanghai, China).

### Oil red O staining and quantitative of porcine granulosa cells

The cells were fixed with 4% paraformaldehyde for 30 min at room temperature, then washed three times with PBS. Adding 300 μL of Oil Red O saturated staining solution (Solarbio, Beijing, China) to each well for 30 min at room temperature, then washed three times with PBS. Take pictures with an inverted microscope. 500 μL of isopropanol was added to each well for extraction of Oil Red O. The absorbance was measured at a wavelength of 510 nm and this absorbance value represents the relative lipid content. There were triplicates per well.

### Ovary, adipose tissue and liver histology analysis

Ovary, adipose tissue and liver were isolated and fixed in 4% paraformaldehyde and maintained at 4 °C until use. Frozen sections were used for Oil Red O staining of ovarian tissue. The fixed adipose tissue, liver and ovaries used to count follicular development were dehydrated and processed for paraffin embedding, and 5 μm sections were stained with H&E. Adipocyte area was measured by Image J (US National Institutes of Health).

According to the following classification method, count the number of follicles in different periods on the ovarian section. Primary follicles were characterized by an enlarged oocyte surrounded by one layer of columnar granulosa cells. Secondary follicles were defined as more than one layer of columnar granulosa cells with no visible antrum surrounding the oocyte and antral follicles were identified as a single large antral space. Atretic follicles were identified as shrinking and absent oocyte and granulosa cells replaced by fibrous material. For detailed steps refer to previous literature [23].

### Vaginal cytology method

A vaginal swab was collected using a pipette tip sucked with 10 μL ambient temperature physiological saline and inserted into the vagina of the restrained mouse. Blowing gently several times and then cells were transferred to a dry glass slide. The slide was air-dried and then stained with approximately 400 μL 0.1% Crystal violet (Sigma-Aldrich, St. Louis, Missouri, USA) for 1 min. The slides were rinsed with water for 1 min and dried in the air, then viewed immediately at 10 × magnification under bright field illumination. The stage of the estrous cycle was determined based on the presence or absence of leukocytes, cornified epithelial, and nucleated epithelial cells. Details were followed according to the previous reference [24, 25].

### RNA extraction and real-time quantification PCR (RT-qPCR)

Total RNA was extracted with RNAex pro reagent (Accurate Biotechnology (Human) Co., Ltd., Changsha, China). The total RNA concentration was measured by DS-11 Spectrophotometer (Denovix, USA) and 500 ng RNA was reversed to cDNA using a Primescript Reverse Transcription kit (Vazyme, Nanjing, China). RT-qPCR analysis of cDNA was performed using SYBR PCR mix (Vazyme, Nanjing, China) on a StepOne Real-Time PCR machine (ABI, Carlsbad, CA). Three repetitions per reaction. Quantitating relative levels of mRNA were calculated using the 2^-∆∆Ct^ algorithm. ACTB was used as an internal reference. Primer sequences used for RT-qPCR are listed in Table 1.

**Table 1:** Primer sequences used in this study (*Sus scrofa*)

Gene name	Forward (5′ to 3′)	Reverse (5′ to 3′)	Size, bp	Accession No.s
*CD36*	TGGGTTAAAACAGGCACGGA	TGCCACAGCCAGATTGAGAA	270	XM_021102279
*SCARB1*	GCTGTTCATCCCCATCGTCT	GGCCTGAATGGCCTCCTTAT	103	NM_213967
*DGAT2*	TCACAGTGGGTCCGAAACTG	GACATCAGGTACTCCCGCAG	260	NM_001160080
*HMGCR*	GCGCTTGCTGTGAGAATGTT	TGCTCTGCAGCCTCTATTGG	146	NM_001122988
*ACAT2*	ACAGTCACCCCAGCTAATGC	TGCTAAAGGAGTAAGCCCGC	102	XM_001928345
*CYP19A1*	TCCGCAATGACTTGGGCTAC	GCCTTTTCGTCCAGTGGGAT	103	NM_214429
*CYP19A3*	AGTGCCCTCGTGCATAAAGT	CGCCACGTTTCTCAGCAAAA	238	NM_214431
*CYP11A1*	GGGCAACCCATTTCCTACCA	CGAGCACTGGTGGTACAGAC	95	KX108746
*StAR*	CGTTTAAGCTGTGTGCTGGG	TCCATGACCCTGAGGTTGGA	132	NM_213755
*ACTB*	TCCCTGGAGAAGAGCTACGA	CGCACTTCATGATCGAGTTG	154	NC_010445

### Western blot analysis

Porcine granulosa cells were washed three times with pre-cooling PBS. 6-well plate were added 150 μL RIPA (Beyotime, Shanghai, China) supplemented with 1% protease inhibitors (AbMole, Chicago, USA) in every well. Scraped the cells off plates on the ice, and collected into a 1.5-mL centrifuge tube and lysed on ice for 30 min, then centrifuged (12,000 r/min) at 4 °C for 10 min. Supernatant protein concentration was determined by BCA protein assay kit (Cwbio, Beijing, China). A 1/4 volume of 5 × loading buffer (Ncmbio, Suzhou, China) was added to an aliquot of the supernatant and boiled for 10 min. 20 μg protein samples from porcine granulosa cells were run on 12% SDS-PAGE gel at 80 V for 4 h, then transferred protein to PVDF membrane (Merck Millipore Ltd., Darmstadt, Germany) at 250 mA for 2 h. PVDF membrane is closed with 5% skim milk for 2 h at room temperature. Finally, the primary antibodies (1:1000) to CD36 (Abways, Shanghai, China), SCARB1 (Abways, Shanghai, China), ACAT2 (Santa Cruz Biotechnology, Dallas, Texas, USA), HMGCR (Santa Cruz Biotechnology, Dallas, Texas, USA), DGAT2 (Abcam, Shanghai, China), CYP19A1(Abways, Shanghai, China), StAR (Cell Signaling Technology, 8449 T, Danvers, Massachusetts, USA), CYP11A1 (Abways, Shanghai, China) and ACTB (Cwbio, Beijing, China) was incubated. The membrane was then incubated with primary antibody at 4 °C overnight and incubate with secondary antibody for 1 h at 4 °C. Secondary antibody (1:3000) against CD36, SCARB1, DGAT2, CYP19A1, CYP11A1 and StAR is HRP goat anti-rabbit IgG (BOSTER, Wuhan, China), against ACAT2, HMGCR and ACTB is HRP goat anti-mouse IgG (BOSTER, Wuhan, China). Finally, the signals were detected by a gel imaging system (Bio-Rad, CA, USA) and analyzed by Image J.

All experiments were repeated at least three times and mean values were derived.

### Statistical analysis

All data were analyzed by the GraphPad prime 8 statistical software. The results were shown as mean ± SEM. One-way ANOVA was used to analyze the difference. Values were considered significant for *P* values less than 0.05 (* = *P* < 0.05; ** = *P* < 0.01).

## Results

### Oleic acid increases lipid accumulation in porcine granulosa cells

**Fig. 1:**
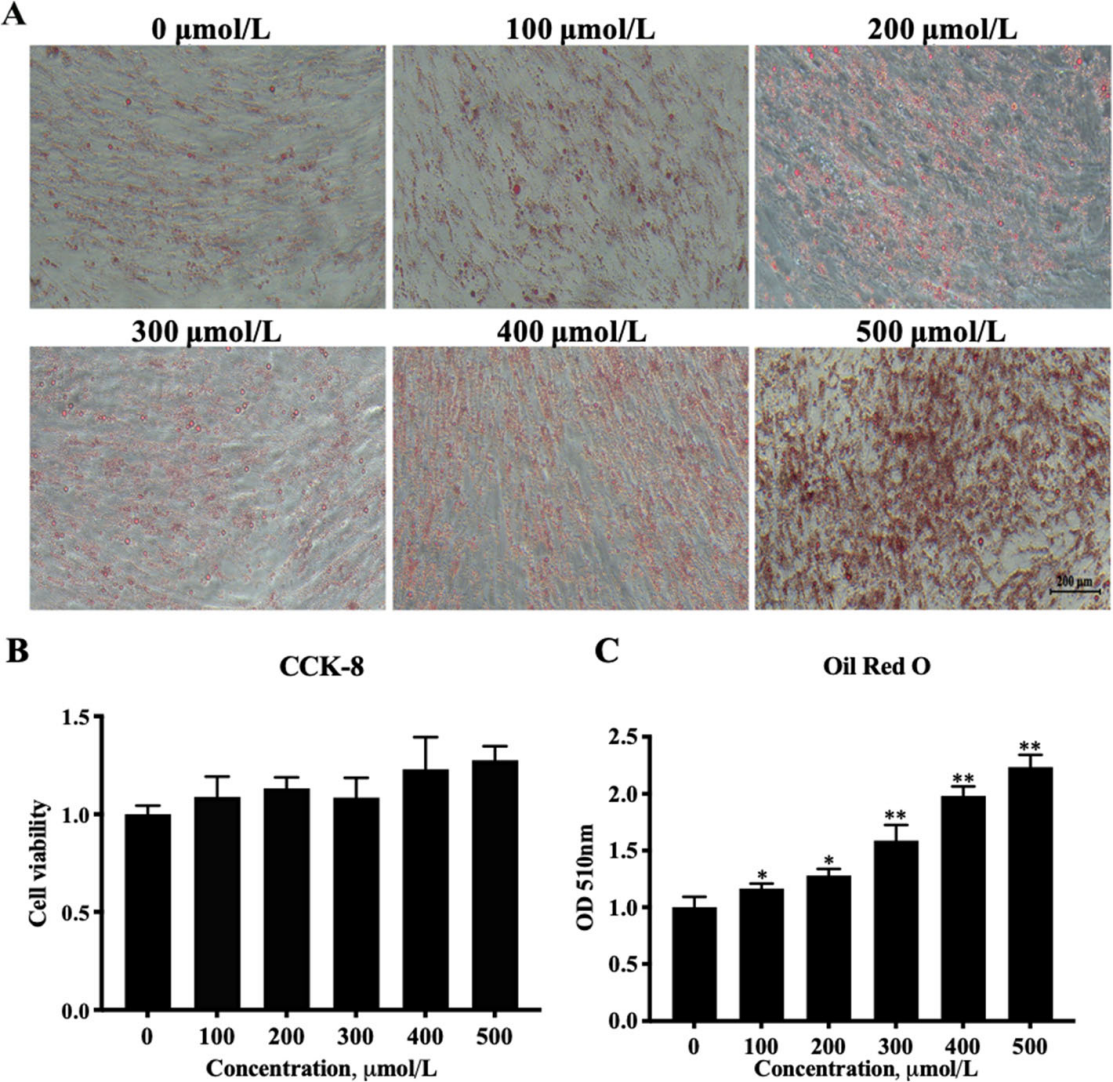
The effects of oleic acid on lipid accumulation in, and the viability of, porcine granulosa cells. The granulosa cells were treated with 0.5% BSA and different oleic acid concentrations or 0.5% BSA as the negative control and harvested after 4 d. **A** Granulosa cells treated with different oleic acid concentrations for 4 d and then stained with Oil Red O. Scale bar = 200 μm; **B** Quantitative analysis of Oil Red O staining; **C** CCK-8 analysis of porcine granulosa cell viability after being treated with different oleic acid concentrations. Note: Data are means ± SEMs of three independent experiments; *P < 0.05, **P < 0.01

Oleic acid induces lipid accumulation in skeletal muscle cells [26]. Consequently, to determine whether oleic acid induces lipid accumulation in porcine granulosa cells, we treated cells with different concentrations oleic acid and stained them with Oil Red O. As shown in Fig. 1A, oleic acid significantly increased the lipid accumulation in a concentration-dependent manner. This was quantitatively shown by the cell absorbance at 510 nm (Fig. 1B). Cell viability was not significantly different after treatments with various sodium oleate concentrations (Fig. 1C). Thus, oleic acid obviously increased lipid accumulations in granulosa cells, but did not affect cell viability.

### Oleic acid increases TG levels and reduces CE levels in porcine granulosa cells

**Fig. 2:**
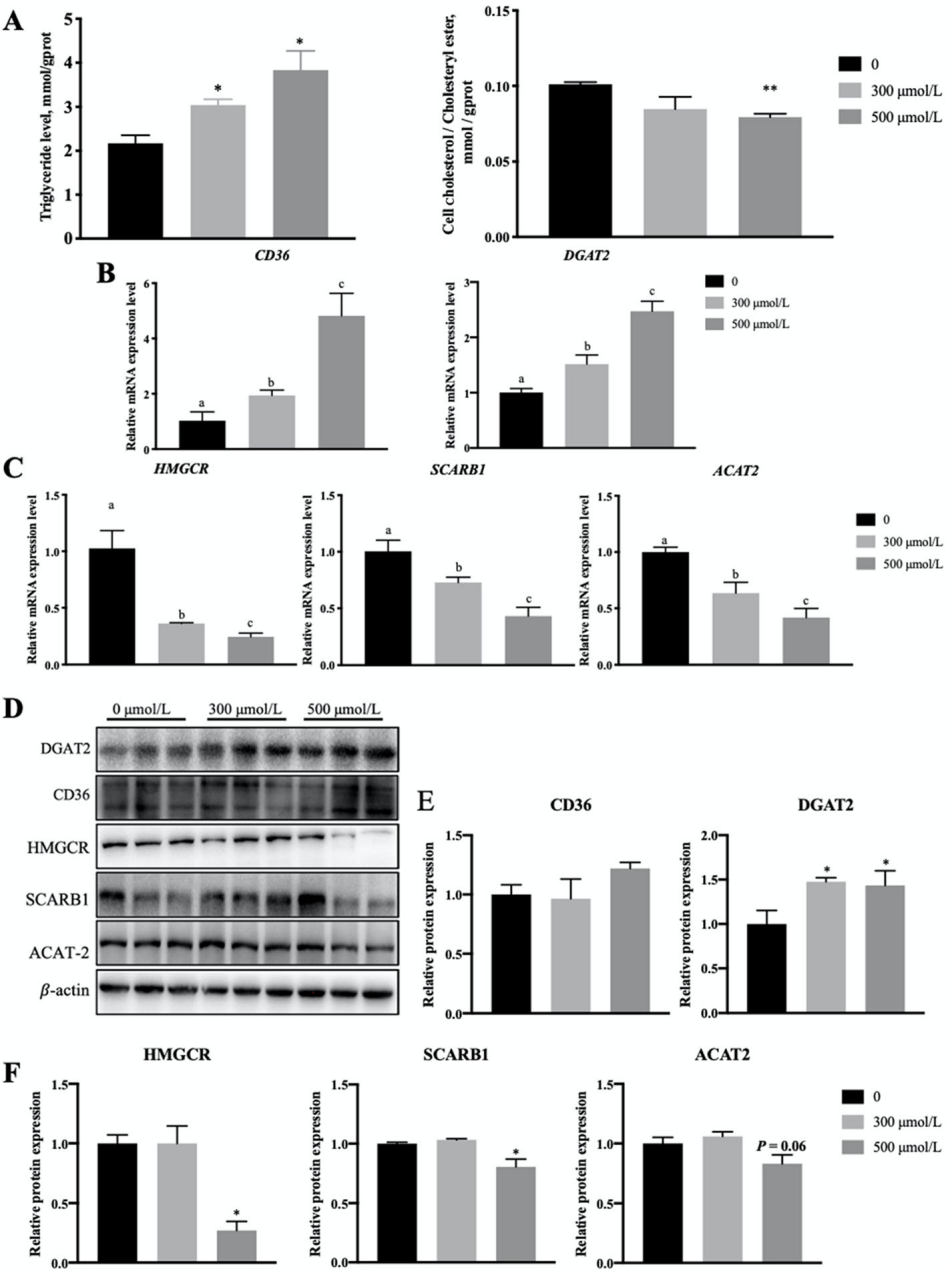
Oleic acid exposure increases triglyceride (TG) levels and reduces cholesteryl ester (CE) levels in granulosa cells. **A** Detection TG and CE levels after independent treatments with 300 μmol/L and 500 μmol/L oleic acid compared with 0 μmol/L (0.5% BSA); **B** RT-qPCR detecting the mRNA expression levels of the fatty acid translocase *CD36* and TG synthase *DGAT2* after oleic acid treatments; **C** mRNA expression of the cholesterol metabolism genes *HMGCR, SCARB1* and *ACAT2* after oleic acid treatments as determined by RT-qPCR; **D** Western blot analysis of TG and cholesterol metabolism-related genes; **E** Quantification of the western blot analysis of CD36 and DGAT*2*; **F** Quantification of the western blot analysis of HMGCR, SCARB1 and ACAT2. Note: Data are means ± SEMs of three independent experiments; *P < 0.05, **P < 0.01

Because oleic acid increased lipid accumulations in granulosa cells, we investigated its effects on lipid types stored in granulosa cells. The main lipids stored in LDs are TGs and CEs. Therefore, we measured their levels. The loading of 300 μmol/L and 500 μmol/L oleic acid obviously increased the TG content (Fig. 2A), but 500 μmol/L oleic acid significantly reduced the CE level (Fig. 2A). Additionally, the expression levels of key genes related to TG and CE synthesis were measured. The mRNA expression levels of the fatty acid translocase *CD36* and diacylglycerol acyltransferase 2 (*DGAT2*) were enhanced after the 300 μmol/L and 500 μmol/L oleic acid treatments (Fig. 2B). Compared with the control group, the mRNA level of 3-hydroxy-3-methylglutaryl-coenzyme A reductase (*HMGCR*), a cholesterol synthesis rate-limiting enzyme, was inhibited in the 300 μmol/L and 500 μmol/L oleic acid groups (Fig. 2C). In addition, the mRNA levels of scavenger receptor class B member 1 (*SCARB1*), which is involved in cholesterol uptake, and acetyl-Coenzyme A acetyltransferase 2 (*ACAT2*), which is involved in CE synthesis, were also reduced after oleic acid treatments, (Fig. 2C). A western blot analysis indicated that 500 μmol/L oleic acid significantly increased the DGAT2 protein level (Fig. 2D, E), but it decreased the HMGCR, SCARB1 and ACAT2 protein levels (Fig. 2D, F). Thus, an increase in the TG level and a decrease in the CE level after oleic acid treatments may result from increases in fatty acid transposition and TG synthesis and decreases in cholesterol absorption and CE synthesis, respectively.

### Oleic acid reduces estradiol and progesterone synthesis in granulosa cells

**Fig. 3:**
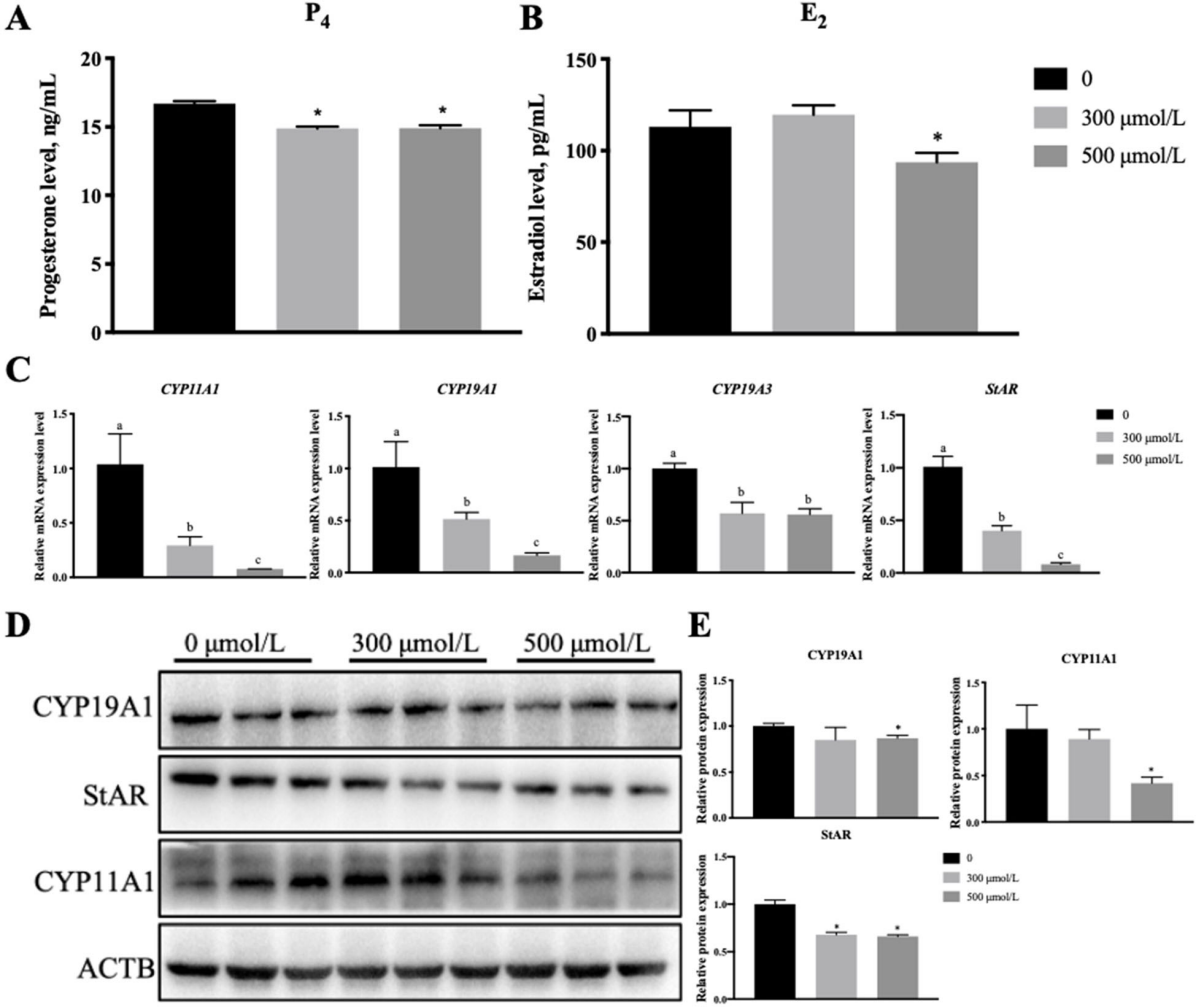
Oleic acid inhibits estradiol and progesterone synthesis in granulosa cells. The cells and media were harvested after 4-day oleic acid treatments. **A** P_4_ levels in media as detected by ELISA; **B** E_2_ levels in media as detected by ELISA; **C** RT-qPCR analysis of steroid hormone synthesis-related genes, including *CYP11A1*, *CYP19A1*and *CYP19A3 and StAR*; **D** Western blot analysis of steroid hormone synthesis-related genes **E** Quantification of the western blot analysis of CYP19A*,* CYP11A1 and StAR; Note: Data are means ± SEMs of three independent experiments; *P < 0.05, **P < 0.01

Because oleic acid reduced the CE levels in granulosa cells, we hypothesized that oleate may affect hormone levels. Granulosa cells mainly synthesize estradiol and progesterone, which play important roles in female reproduction [27, 28]. ELISA results showed that the progesterone (P_4_) and estradiol (E_2_) levels decreased in granulosa cells treated with oleic acid (Fig. 3A, B). RT-qPCR and western blot analyses also showed that oleic acid significantly inhibited the expression of enzymes related to steroidogenesis, including cytochrome P450 family 19 subfamily A member 1(CYP19A1) and 3 (CYP19A3), CYP11A1 and steroidogenic acute regulatory protein (StAR) at the mRNA (Fig. 3C) and protein (Fig. 3D, E) levels. Oleic acid reduced the progesterone and estradiol levels of porcine granulosa cells.

### The effects of oleic acid on lipid accumulation in mice ovarian tissues

**Fig. 4:**
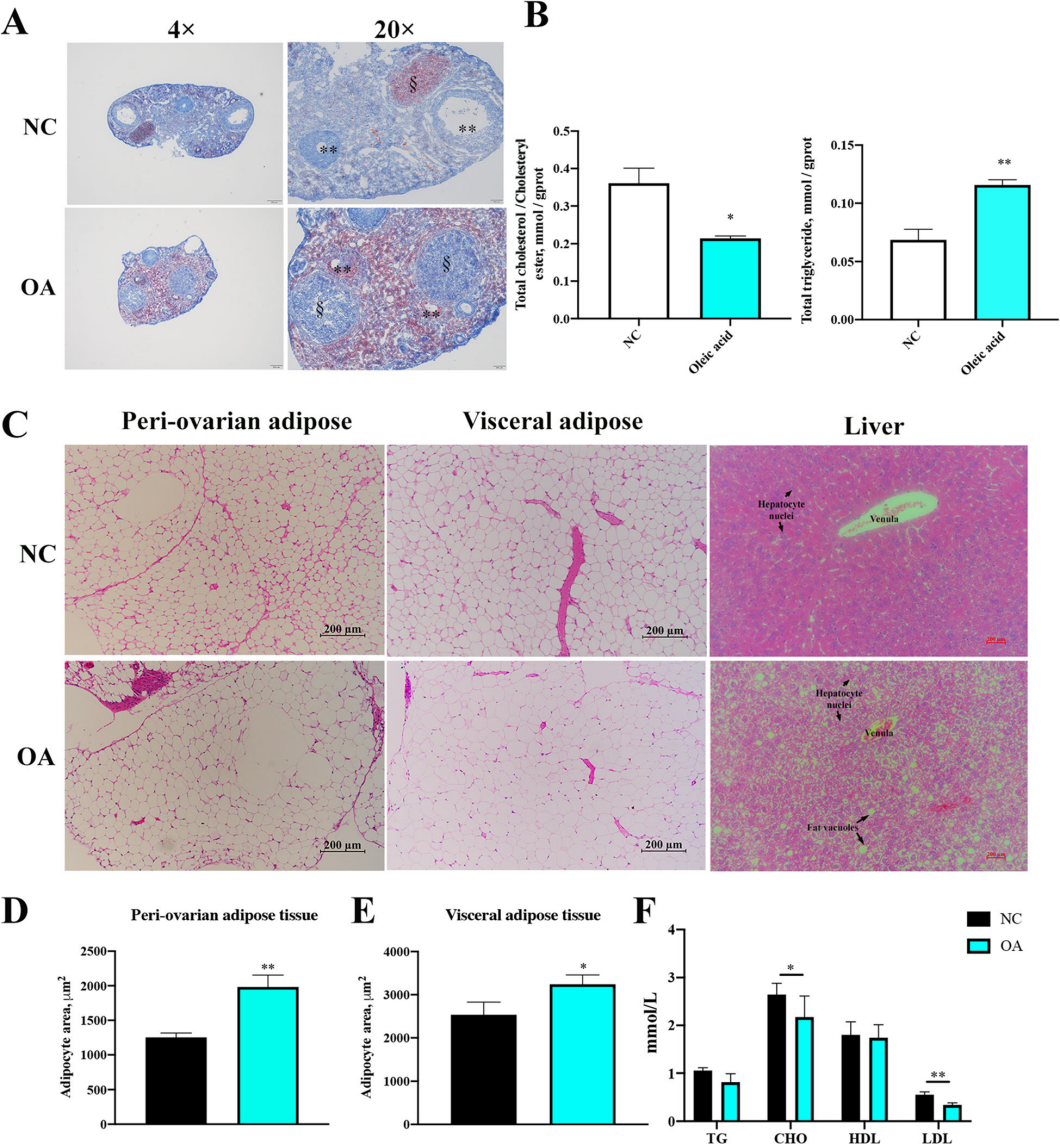
Oleic acid increases triglyceride (TG) levels and reduces cholesteryl ester (CE) levels in mice ovarian tissues. The female mice were fed with a 20%-oleic acid or negative control diet (NC) for 8 weeks, and tissues were dissected after the animals were sacrificed. **A** Oil Red O staining of ovarian tissues (*n* = 3), scale bars = 100 μm and 200 μm; **, antral follicle; §, corpora lutea. **B** Detection of the TG and CE levels in the ovaries (*n* = 5); **C** Representative images of H&E-stained sections of peri-ovarian adipose, gonadal adipose and liver tissues, scale bar = 200 μm; **D** Adipocyte area statistics of peri-ovarian adipose tissue (*n* = 5); **E** Adipocyte area statistics of gonadal adipose tissue (*n* = 5). **F** The sera of female mice were collected to determine the statistics of TG, CE, high-density lipoprotein (HDL) and low-density lipoprotein (LDL) levels (*n* = 5). Note: Data are represented as the means ± SEMs. Significance was determined using Student’s *t*-test, *P < 0.05, **P < 0.01

To further clarify whether oleic acid affects female reproduction, we added 20% oleic acid to the female mouse diet. Morphologically, the Oil Red O showed that oleic acid increased the lipid accumulations in ovaries (Fig. 4A). Additionally, as shown in Fig. 4B, the TG level increased, while the CE level decreased in ovaries of the oleic acid-fed group. The HE staining showed that oleic acid also increased the areas of gonadal and peri-ovarian adipose cells, as well as the lipid accumulation in the liver (Fig. 4C). Further quantification of the adipocyte areas confirmed the increased size of adipocytes in the oleic acid-fed group (Fig. 4D, E). In addition, lower total cholesterol and low-density lipoprotein serum levels were detected after oleic acid treatments (Fig. 4F). Unexpectedly, the serum TG level did not show an upward trend in the oleic acid-fed group (Fig. 4F).

### The effects of oleic acid on hormone secretion and follicular development in female mice

**Fig. 5:**
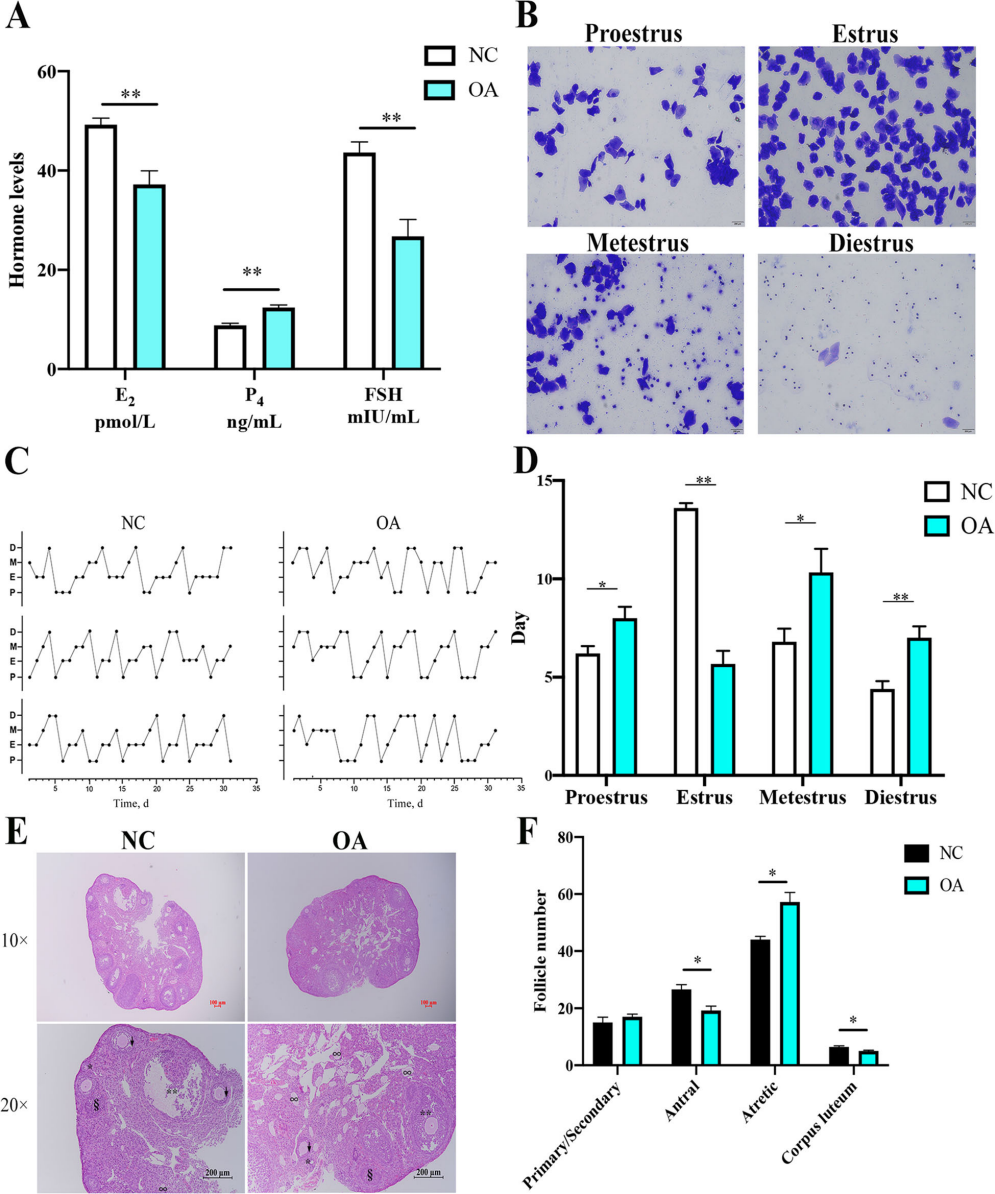
The effects of oleic acid on hormone secretion and follicular development in female mice. **A** The serum of female mice were collected to determine the E_2_, P_4_ and FSH levels (*n* = 5); **B** Representative image of vaginal smear staining to detect the estrus cycle, scale bar = 200 μm; **C** and **D** Statistics of various stages of the estrus cycle (*n* = 5); **E** Representative images of H&E-stained sections of an ovary, scale bars = 100 μm and 200 μm; **F** Statistics of the follicle numbers during various developmental periods (*n* = 5), *, primary follicle; ↓, secondary follicle; **, antral follicle; §, corpora lutea; ∞, atretic follicle. Note: Data are represented as the means ± SEMs. Significance was determined using Student’s *t*-test, *P < 0.05, **P < 0.01

Owing to the CE level decreased in the oleic acid-fed mouse group, we investigated the effects of oleic acid on serum hormone levels. As shown in Fig. 5A, the E_2_ level decreased, which was consistent with the detected levels in porcine granulosa cells. On the contrary, the P_4_ level increased in the sera of the oleic acid-fed group members (Fig. 5A). In addition, the levels of the gonadotropin FSH in the sera were also lower after feeding oleic acid (Fig. 5A). Compared with the control, the estrous cycle was disordered in the oleic acid-fed group, with a shortening of the days of estrus (Fig. 5B-D). Moreover, in the oleic acid-fed group, the numbers of antral follicles and corpora lutea were relatively depleted, whereas the numbers of atretic follicles were obviously increased (Fig. 5E, F). Thus, oleate significantly decreased the estradiol level, the estrus days and numbers of antral follicles and corpora lutea, while it increased the numbers of the atretic follicles.

## Discussion

**Fig. 6:**
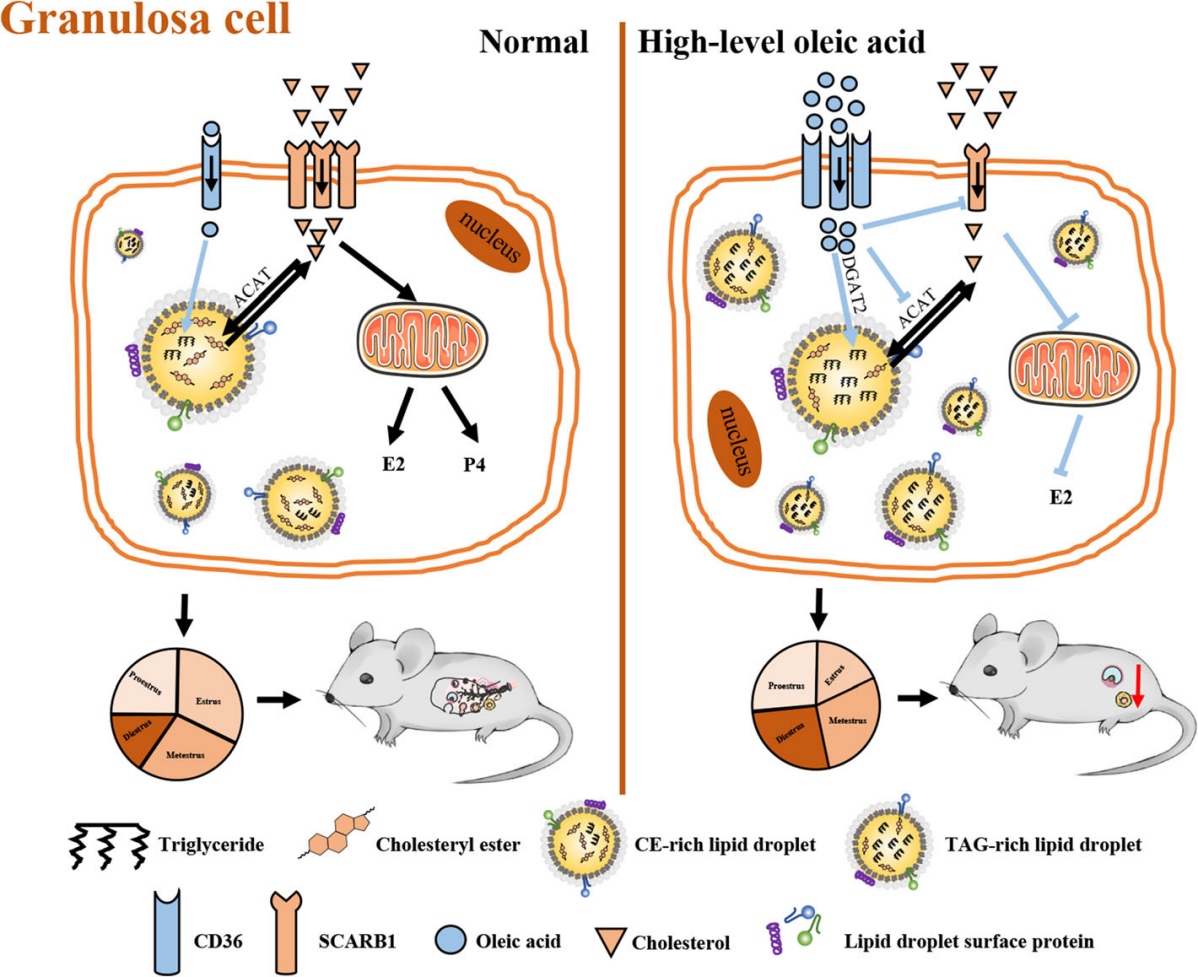
Schematic summary of oleic acid reducing steroidogenesis by changing the lipid type stored in lipid droplets of ovarian granulosa cells. Note: Red arrows indicate the promotion of a given process; Blunt heads indicate that a process is inhibited

In our study, we found that oleic acid plays a negative role in steroid hormone synthesis by changing the lipid type stored in ovarian granulosa cells. Specifically, oleic acid promoted TGs accumulation and reduced the CEs level (Fig. 6). Our finding provides new insights into the effects of oleic acid on steroidogenesis.

Oleic acid is a monounsaturated omega-9 fatty acid and is always regarded as a beneficial non-esterified fatty acid [29]. Indeed, Oleic acid supplementation leads to TGs accumulation and decreases lipid toxicity [30]. However, it is also an abundant free fatty acid in the blood and follicular fluids of livestock that are in a negative energy-balance state [31, 32]. A high level of oleic acid may have detrimental effects on female reproduction and fertility. Here, we found that oleic acid increased lipid, mainly TG accumulation and reduced CE accumulation, which led to decreased estradiol and progesterone production. Steroidogenesis is a complex process in which cholesterol is transported to mitochondria and converted into steroids through a series of enzymatic steps. Therefore, decreases in the cholesterol levels of granulosa cells may lead to lower steroid hormone levels [21].

In skeletal muscle cells, 500 μmol/L oleate, as an inducer of lipid deposition, increases lipid accumulation and has no effect on cell viability [26]. Initially, we also found that oleic acid increased lipid accumulation and had no effect on cell viability. Consistent with our results, the addition of 400 μmol/L oleate to bovine granulosa cells also induces LD accumulation and has no effects on apoptosis and cell proliferation [19]. In our study, the analysis of relative gene expression levels indicated that oleic acid significantly up-regulated the transcription and translation levels of the fatty acid transporter *CD36* and triglyceride synthase *DGAT2*. Oleic acid also increases the transcript levels of the fatty acid transporters *CD36* and solute carrier family 27 member 1 in bovine granulosa cells [19]. Adding either saturated (palmitic and stearic) or unsaturated (oleic and linoleic) fatty acids to the medium of bovine mammary epithelial cells stimulated TG accumulation in a concentration-dependent manner from 50 to 400 μmol/L, as well as the expression of *CD36* mRNA [33]. Oleic acid also promotes cervical cancer progression by up-regulating *CD36* expression [34]. The inhibition of *CD36* leads to a reduction in LD formation [35]. This result indicated that oleic acid increases the TG content by increasing the absorption and incorporation of fatty acids. However, the exact regulation mechanism of oleic acid on *CD36* expression remain unclear and are worth further analysis.

In addition, our results showed that oleic acid increases TGs accumulation while reducing the CEs level. Cholesterol is the raw material for steroid synthesis and is derived from a variety of sources, including lipoprotein uptake, endogenous cholesterol synthesis and release from LDs stored in cells [21]. In cholesterol biosynthesis, *HMGCR* is a rate-limiting enzyme [36, 37], and *SCARB1* is high-density lipoprotein receptor that allows cholesterol uptake [38, 39]. The enzyme *ACAT2* is responsible for the formation of sterol esters, which are involved in an important mechanism for controlling steroidogenesis rates in gonadal tissues by modulating substrate availability [40]. The gene expression analysis showed that oleic acid inhibited the mRNA and protein levels of ACAT2, HMGCR and SCARB1, which suggested that oleic acid reduced the cholesterol level by repressing its absorption and synthesis. Consistent with our results, oleic acid reduces cholesterol synthesis by inhibiting *Hmgcr* expression in C6 glioma cells [41]. More importantly, 400 μmol/L oleate induces the down-regulation of the granulosa cell identity marker *FOXL2* and the up-regulation of the Sertoli cell marker SRY-box transcription factor 9 in bovine granulosa cells [20]. In a bovine granulosa cell-culture model, *α*-linolenic acid and *cis*-9, *trans*-11-conjugated linoleic acid up-regulated *CD36* expression and down regulated *FOXL2* expression. Both fatty acids significantly down-regulated the *StAR, CYP19A1*, *FSHR* and *LHGCR* expression levels and decreased estradiol and progesterone production [42]. Thus, the TG accumulation in granulosa cells after an oleic acid treatment may lead to changes in the characteristics and functions of granulosa cells.

Here, the ELISA analysis indicated that 500 μmol/L oleic acid obviously reduced the estradiol and progesterone levels though inhibiting the expression of *CYP11A1*, *CYP19A1, CYP19A3* and *StAR*. Previous studies also found that oleic acid significantly reduces progesterone and estradiol levels by inhibiting the expression of key genes linked to gonadotropin signaling, such as *FSHR* and *LHGCR* (Luteinizing Hormone/Choriogonadotropin Receptor), and steroidogenesis, such as *StAR, CYP11A1, 3β*-*HSD* and *CYP19A1* in bovine granulosa cells [19]. But unlike other species present only one functional genomic copy referred to as *CYP19A1,* the pig genome contains three *CYP19* homologs copies, referred to as *CYP19A1*, *CYP19A2* and *CYP19A3* [43]. Among them, *CYP19A1* is mainly expressed by porcine blastocysts during implantation, *CYP19A2* is identified in placental tissue. However, only transcripts of the *CYP19A3* paralog is predominantly expressed in the ovary [44]. Nucleic acid sequence analysis showed that the similarity between *CYP19A1* and *CYP19A3* reach to 93%, which may lead to primers to amplify *CYP19A1* but amplify *CYP19A3* at the same time. These results may mislead researchers to think that *CYP19A1* is mainly expressed in the pig ovary but not *CYP19A3*. In present study, we detected the mRNA expression of *CYP19A1* and *CYP19A3* and found both were repressed after oleic acid treatment. In mouse Leydig cells, oleic acid also reduces testosterone synthesis [22]. Therefore, oleic acid decreased the cholesterol levels of steroidogenic cells which may lead to lower steroid hormone levels.

The fatty acid contents in follicular fluids are increased when high-yielding dairy cows are in a negative energy-balance state [45], which is harmful to reproductive functions. Sexually mature female Ossabaw pigs have been fed high fat/cholesterol/fructose as a model to study the effects of obesity on female reproduction, and abnormal metabolic syndrome, dysfunction of estrous cycles and hormone secretions were found in these obese pigs [46]. Thus, energy metabolism disturbances may affect hormone secretion. In previous study, oleic acid was injected into the dominant follicles of heifers in situ by ultrasound-guided injection approach. Interestingly, the oleic acid injected animals showed reduced ovulation rates and reduced production of E_2_ hormone compared to that of the control group [47]. However, studies on the correlations between an oleic acid-rich dietary and fertility are limited in females. In the present study, we used a mouse model to explore the effects of high levels of dietary oleic acid on ovarian functions, including hormone secretion, estrous cycle and follicular development. Ovarian granulosa cells are an important kind of follicular cell, and they secrete sex hormones, such as estrogen and progesterone, responsible for regulating female characteristics, the menstrual–ovulation cycle and pregnancy [48]. Consistent with porcine granulosa cell results, oleic acid increased the TG contents and reduced the CE levels of female mice ovaries, which decreased estradiol production, caused an estrous cycle disorder, and reduced the numbers of antral follicles and corpora lutea. The hypothalamic-pituitary-ovarian axis describes a regulatory system in which pituitary gonadotrophins stimulate ovarian folliculogenesis and the production of steroid hormones, which in turn exercise feedback control on the production of pituitary gonadotrophins [49]. The P_4_ level increased in the serum of the oleic acid-fed group members, which may improve the negative feedback to FSH and lead to the decline of FSH production. An oleic acid-rich diet reduces cholesterolemia, while promoting LXR-dependent hepatic lipogenesis [50]. Our results also showed that oleic acid reduced total cholesterol and low-density lipoprotein serum levels. In hence, high-level oleic acid in serum or follicle fluid probably exerts a deleterious role in reproduction competence result from a decline of steroid hormone synthesis.

## Conclusions

Although oleic acid does not produce a lipotoxic effect similar to that of saturated fatty acids, it changes the characteristics and functions of granulosa cells and reduces the secretion of steroid hormones, which have deleterious effects on female reproduction. More importantly, the effects of cholesterol metabolism on steroidogenesis in ovarian granulosa cells was explored and may provide a new strategy for the treatment of sterility-related steroid hormone disorders.

## Data Availability

The data sets used and analyzed during the current study are available from the corresponding author on reasonable request.

## Abbreviations

StAR: Steroidogenic acute regulatory protein
CYP11A1: Cytochrome P450 family 11 subfamily A member 1
CYP19A1: Cytochrome P450 family 19 subfamily A member 1
CYP19A3: Cytochrome P450 family 19 subfamily A member 3
DGAT2: Diacylglycerol Acyltransferase 2
HMGCR: HMG-CoA reductase
SCARB1: Scavenger receptor class B member 1
ACAT2: Acetyl-Coenzyme A acetyltransferase 2
FOXL2: Forkhead box L2
LHGCR: Luteinizing Hormone/Choriogonadotropin Receptor
LDs: Lipid droplets
TG: Triacylglycerol
CE: Cholesteryl ester
HDL: High-density lipoprotein
LDL: Low-density lipoprotein
